# Mood regulation in bipolar disorders viewed through the pendulum dynamics concept

**DOI:** 10.1186/s40345-014-0009-6

**Published:** 2014-06-17

**Authors:** Elias Koutsoukos, Elias Angelopoulos

**Affiliations:** Signal Processing Laboratory, University Mental Health Research Institute, 2 Soranou Efessiou, Athens, 115 27 Greece; Medical School, 1st Psychiatric Dept., Eginition Hospital, Athens University, 72-74 Vas. Sofias Ave., Athens, 115 28 Greece

**Keywords:** Bipolar disorders, Mood oscillations, Feedback control, Pendulum dynamics, Lithium, Anti-convulsants, Mood stabilizers

## Abstract

Bipolar disorders have been characterized by powerful fluctuations of energy, mood, and thinking patterns. Mood episodes (manic or depressive) could be considered as deviations of a psycho-physiological index above or below a conventionally defined value called ‘normothymia’. In the present study, we analyzed the feedback techniques used to suppress the oscillatory activity exhibited on an inverted pendulum device. Subsequently, we examine the degree that this multimodal feedback design could be considered on a hypothetical pendulum where the mood plays the role of the suspended mass, and the force balance compensation circuitry is substituted by drug-specific therapeutic interventions. The study does not concern a model of bipolar illness that could simulate numerically various phases of mood episodes but focuses on the functional similarities regarding the correction treatments applied on the two different oscillating systems giving a potential perspective of how techniques of feedback control may enhance the conceptualization of the treatment schemes followed in recent guidelines for biological treatment of bipolar disorders. Our theoretical consideration, along with observations on clinical level, gives support to the concept that the compensation of the mood oscillations should be adaptive with selective therapeutic interventions that compensate the excited system in different time scales.

## Background

Bipolar disorder (BD) is a complex illness, involving the dysregulation of mood, sleep, cognition, endocrine, and motor systems. A true understanding of the pathophysiology of this disorder must encompass different systems such as: molecular, cellular, and behavioral together with the strong interactions that exist in their underlying mechanisms. Decades of research in this disorder have identified abnormalities in neurotransmitter systems, and there is a growing appreciation that signal transduction pathways play a crucial role in mediating the dysfunction in multiple neural network systems and physiological processes (Bhalla and Iyengar 2007). Complex signaling networks are undoubtedly involved in regulating such diverse functions as mood, appetite, and wakefulness and therefore are involved in the pathophysiology of mood and vegetative symptoms. Furthermore, there is clear evidence that signaling pathways are targets of the most effective pharmacologic treatments for bipolar illness (Chepenik et al. 2010). Recent therapeutic approaches consider lithium as the mainstay of bipolar disorder pharmacotherapy for acute mood episodes, switch prevention, prophylactic treatment, and suicide prevention. Its effectiveness in treating BD has been associated with significant neurotrophic and neuroprotective properties. Direct targets of lithium involved in these neurotrophic/neuroprotective effects include the phosphoinositol (PI) cycle (Berridge et al. 1989), the protein kinase C (PKC), and mitogen activated protein kinases (MARCKS) pathways (Manji et al. 1993; Chen et al. 1994; Hahn and Friedman 1999; Seelan et al. 2008; Manji and Lenox 1999), neurotrophins, glycogen synthase kinase 3 (GSK3) (Gould et al. 2004; Jope and Bijur 2002; Klein and Melton 1996), and mitochondrial/endoplasmic reticulum key proteins (Warsh et al. 2004). Moreover, the initial effects of anticonvulsants involve regulation of the glutamate excitatory neurotransmission (Loscher 1993; Collins et al. 1994; Smith and Meldrum 1995) and/or gamma aminobutyric acid (GABA) inhibitory neurotransmission (Gram et al. 1988; Nilsson et al. 1990). Similar to lithium, intracellular mechanisms of anticonvulsants, primarily valproic acid (VPA) and carbamazebine (CBZ), include regulation of several protein kinase signaling pathways (Manji et al. 1999; Lewin and Bleek 1977; Xiaohua et al. 2002) leading to regulation of gene expression. Common genes that can be regulated by mood stabilizers are more likely to be the final normalizing components in BD.

## The introduction of energy and thermodynamic models in bipolar illness

Bipolar disorders have been characterized by powerful fluctuations of energy, mood, and thinking patterns, providing a unique frame to develop and test psycho-dynamic theories that involve mechanisms of biological homeostasis and regulation. Recognizing that psychological phenomena are biological processes, Sigmund Freud achieved the seminal insight of viewing them as primarily energetic and developed his psychodynamic theory on the basis of thermodynamics (Basch 1988). The approach was mainly influenced from the closed systems thermodynamics, where feedback processes maintain the equilibrium state of the system. Under this view, bipolar swings represent a failure of psychobiological feedback mechanisms to maintain homeostatic equilibrium. Fischer (1975) presumed that biogenic amine imbalance theories also imply a failure of this homeostatic regulation. According to a different angle of view, bipolarity could be generated by an exaggeration of normal biological rhythms (Wehr and Goodwin 1979; Wirz-Justice and Wehr 1983). Thus, the current considerations for the bipolar illness are the homeostatic model implicit in Freud's psychodynamics and most neuroamine deficit/excess theories and the oscillatory model of exaggerated biological rhythms. Sabelli et al. (1990) introduced a thermodynamic model of bipolarity that includes both homeostatic and oscillatory features and adds the most important feature of open system thermodynamics: the criterion of novel structures in bifurcation processes. They proposed that bipolarity is the result of exaggerated biological energy that augments homeostatic oscillatory and creative psychological processes.

The temporal pattern of mood in BD has received research interest, especially under the influence of new scientific perspectives of the nonlinear dynamical analysis. Results from studies based on long-term daily mood records obtained from patients with BD and normal subjects indicate that long-term mood in BD, although not cyclic, is still highly organized compared with that of normal controls and can be characterized by the presence of a low-dimensional chaotic attractor (Gottschalk et al. 1995). Bipolar disorders, in general, are characterized by recurrent, alternating episodes of mania and depression. According to a recent theoretical study (Goldbeter 2011), the tendency to mania and depression is correlated with the activity of two hypothetical neural circuits that promote, respectively, the manic or the depressive state, by inhibiting each other. The proposed model can generate fundamental periodic oscillations, complex shapes with unequal durations, or small amplitude oscillations around one of the two states preceding large amplitude periodic changes in the propensities to mania or depression. The nonlinear mathematical oscillators have received attention in a number of research works that involved this theoretical background in the study of BD. Daugherty et al. (2009) investigated the generation, maintenance, and interaction of bipolar states using numerical simulations based on two nonlinear oscillating models. In the same context, Nana (2009) modeled the periodic mood variations of a bipolar II patient with a negatively damped harmonic oscillator. In the field of nonlinear biochemical reaction equations, Frank (2013) introduced two signaling pathways leading to the activation of two enzymes that play a key role for cellular and neural processes in a nonlinear limit cycle model for the oscillatory mood variations as observed in patients with cycling bipolar disorder.

On the other hand, Salerian (2010) suggested that brain function is region-specific and governed by complex system dynamics and thermodynamic laws and hence, any change in brain homeostasis (temperature, neurotransmission, or content) causes brain dysfunction.

Although the dynamics of bipolar illness are complex and the underlying mechanism could exhibit either linear or nonlinear behavior, the therapeutic approach as a correction mechanism of the mood oscillations should be adaptive and flexible in order to encounter the complex profile of the illness.

In the present study, we consider the physics of an inverted pendulum compensated by a force balance topology, with a theoretical pendulum where the mood plays the role of the suspended mass and the force balance compensation circuitry is replaced by drug-specific therapeutic interventions. In both systems, the oscillating members (mass/mood) are free to swing in response to any external or internal excitation, while the correction mechanism restores the equilibrium of the system. According to this view, the action of feedback interacts continuously with the oscillating medium by compensating the oscillations completing, in this way, a closed loop. The purpose of the present work is not to simulate, in a manner, the generation of mood oscillations observed in BDs neither to directly associate one by one the functional properties of an electromechanical pendulum system (EPS) with those of a mood pendulum system (MPS) but to found functional commonalities between the feedback treatment applied on the experimental pendulum device and the medical manipulations regarding the treatment of mood episodes. Additionally, in the present study, introduced topics of feedback control, such as the velocity-type compensation, time-selective regulation, and stability, have been co-examined with the treatment manipulations followed in the guidelines for biological treatment of bipolar disorders.

## Methods

### Stabilization of the inverted pendulum

The force balance principle has been proven as a dominant compensation topology for the stabilization of moving elements in sensors and other assemblies where motion control is critical. The generalization of this approach has also been applied in the modeling of complex physical phenomena and in the understanding of correction procedures and manipulations in economic problems (Luenberger 1975; Sengupta 1997). For example, recent studies (Koutsoukos and Melis 2005, 2007) in the field of ground motion (seismic) instrumentation have shown that novel seismic instrumentation topologies following the application feedback were extremely sensitive, linear, and stable enough to detect both strong and long period external disturbances. In both the abovementioned works, the pendula assemblies were ideal experimental paradigms to study the properties and the dynamics of feedback control on sensitive devices under observatory grade conditions.

**Figure 1:**
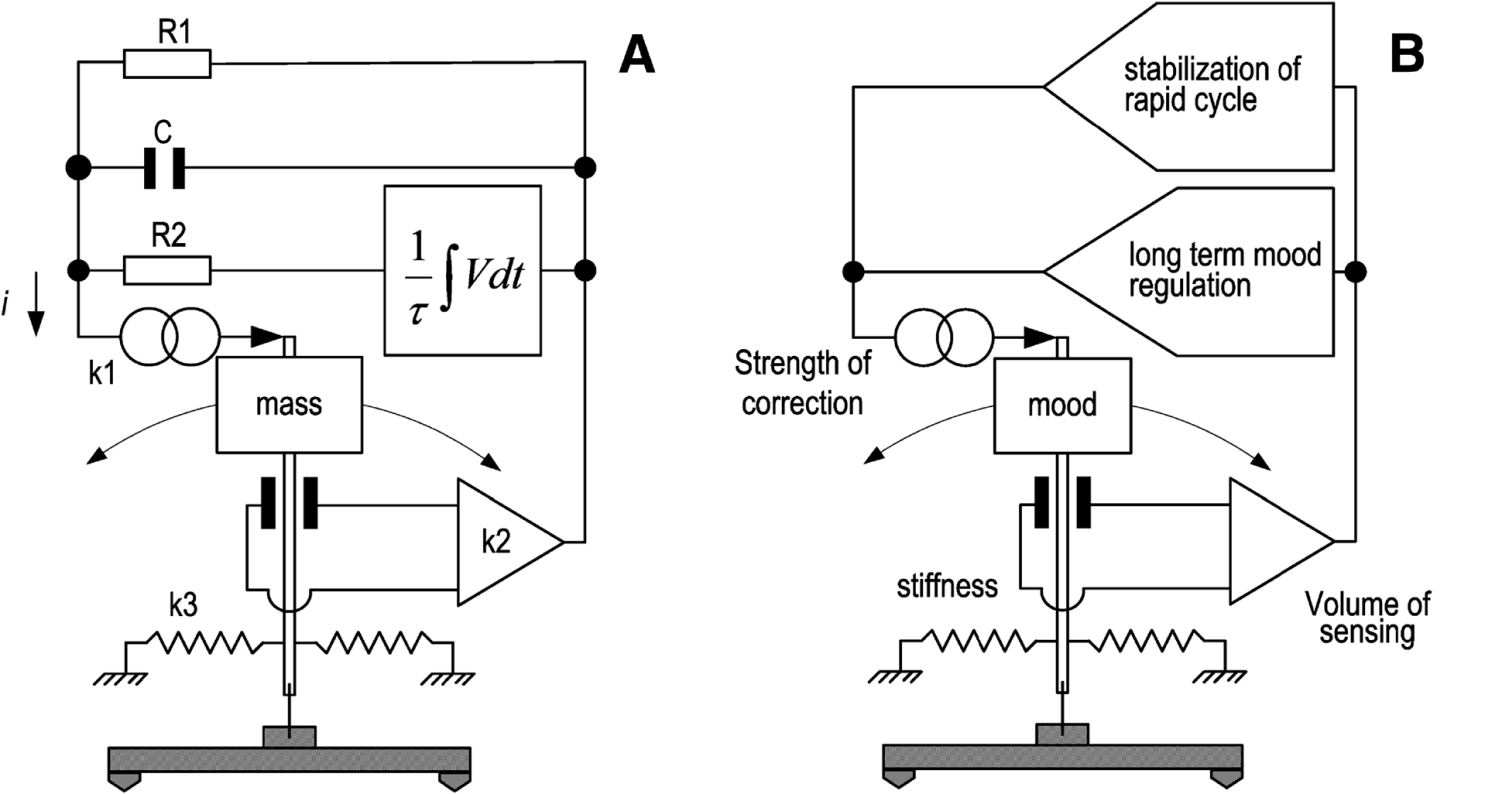
**Description of the mass-pendulum arrangement. (A)** An electromechanical pendulum system supported by a force balance topology that maintains the mass *M* position stable in the equilibrium position. Nodes including *R*_1_ and *C* serve the recovery of the mass position following fast disturbances of the system, while integrator *τ* and the in-series resistor *R*_2_ ensures the long-term stabilization of the mass. *k*_3_ is the stiffness constant of the system, and *k*_2_ is the constant that defines the gain of the device that senses the deviation of the pendulum around the equilibrium position. *k*_1_ is the force constant (motor constant) which defines the amount of the applied correction in response to the external excitations of the system. **(B)** In analogy, a mood pendulum system is illustrated where the mood plays the role of the mass seen in the previous block and the feedback nodes are substituted by analogous ‘therapeutic actuators’ that keep the mood stable. The upper node compensates the fast and transient mood episodes (mood oscillations) and usually is implemented by the use of some antiepileptic drugs. The lower node exhibits integrative behavior and compensates mood episodes with long time evolution acting as long-term mood stabilizer. Usually, this type of regulatory response is served by the action of lithium.

The description of the mass-pendulum arrangement and a reference to the principles of the closed loop control is essential for the later coupling of the two systems. Figure 1A illustrates an inverted pendulum with the mass *M* suspended in vertical position via a flexure on the solid base of the instrument. The mass *M* (inertial mass) is free to swing bilaterally to the resting position, while the feedback counterbalances this motion. Electrical signals corresponding to the kinetic changes of the mass *M* are amplified and fed back through the electromagnetic actuator to the mass via a multiple frequency-dependent path, thus closing the servo loop. Equation 1 shows the three different contributions that form the total current *i*(*t*): (a) the proportional current through the resistor *R*_*1*_, which practically damps the low-frequency oscillation of the system occurring at the lower frequency corner of the closed loop (at this frequency, the differential and integral feedback fail to maintain the equilibrium of the system because they have opposite phase and cancel each other), (b) the differential current through the capacitor *C* (the voltage across the capacitor is proportional to ground velocity), and (c) the integral current through the resistor *R*_*2*_, which is derived from the integration of the output voltage *V* with a time constant *τ* (this current compensates the semi-permanent or long-period disturbances of the mass).

1 $$ i(t)=C\frac{ dV}{ dt}+\frac{V}{R_1}+\frac{1}{R_2\tau }{\displaystyle \int Vdt} $$

Expressions 2 and 3 define in Laplace domain the transfer function of the whole system regarding the output *V(s)* in response to the external motion *X*_*c*_*s*.

2 $$ \left[ Cs+\frac{1}{R_1}+\frac{1}{R_2\tau s}\right]V(s)=-\frac{M{s}^2}{k_1}\left[\frac{1}{k_2}V(s)+{X}_c(s)\right] $$

3 $$ \frac{V(s)}{X_c(s)}=\frac{\frac{-M{s}^2}{k_1C}}{\frac{M}{k_1{k}_2C}{s}^3+{s}^2+\frac{1}{R_1C}s+\frac{1}{R_2 C\tau}} $$

where *M* = 0.25 kg is the mass of the pendulum, *k*_1_ = 15 N/A is the motor constant needed for the recovery of the mass deflection, *k*_2_ = 200,000 V/m is the displacement constant (it defines the whole sensitivity of the device), *R*_1_ = 700,000 Ω is the resistance of the proportional path, *R*_2_ = 470,000 Ω is the resistance of the integral path, *C* = 20 μF is the capacitance of the derivative path and *τ* = 80 s is the integrator constant. Following the substitution of the above values, the transfer function (3) describes the operation of a closed loop system that restores the mass position in response to any external disturbance. The stability of such a controlled system should be examined for failures nested inside the loop. In the present study, the impulse response of the system has been used for examining the stability of the inverted pendulum device under operational, as well as, computational level. The impulse response describes the reaction of a dynamic system in response to an impulse excitation of the input. Consecutively, Nyquist plot (Nyquist 1932) has been used as method to graphically ascertain the stability margins of EPS closed loop. The previous procedure mentioned defines the critical conditions of the system before instability sets in. Such experimentations are useful since the extracted results could be considered in cases where the stability of the mood oscillating system is examined.

### Cross description of the mass-mood pendulum arrangements

The cross description of EPS and MPS seen in Figure 1 contributes towards better understanding of the functional similarities of the specific elements that act in different substrates: the electromechanical and the socio-psycho-biological. In analogy, regarding the oscillating members, the oscillating mass in EPS is compared with the mood in MPS that swings bilaterally to the equilibrium point (normothymia), with the relative poles being mania and depression, respectively. The parameter *k*_3_ refers to the spring constant or the stiffness of the suspension flexure in the EPS. The resilient properties of this flexure allow the mass to restore the null position and to oscillate at a defined natural period (*ω*_*0*_) with a damping (*h*), (Koutsoukos and Melis 2005). Also, stiffness defines the excitability or the sensitivity of the oscillating member. All these have effect in the case of an inverted pendulum without feedback control, where the pendulum terminates the oscillation after a time defined mainly from its damping. In analogy, this stiffness may refer to the sensitivity of a person to initiate normal mood fluctuations that are usually compensated with physiological mechanisms. In our study, we consider the existence of pathological bipolar background where the mood changes are severe and impose medical assistance and so medical treatment correction. Under this situation where the feedback dominates parameter *k*_3_ has no effect in the functioning of EPS since the resilient properties of the suspension have been substituted by the broadband force balance correction. This is the reason that *k*_3_ is not participated in the formation of the transfer function. In analogy in the level of MPS, the medication is present and the medication feedback dominates as regulatory mechanism.

The device *k*_*2*_ that senses the error signal in EPS (usually an electronic amplifier and the assorted filters), corresponds in MPS to an envelope of information acquired from the clinical estimation of the mood state (inter-subject interaction by means of therapist-patient interaction, appearance, social behavior, information from relatives, and application of diagnostic criteria). The role of this sensing is crucial for both models. Over or under estimation of the error information leads to subsequent miscorrection and generalized nonpredictable instabilities. Since the challenge is to stabilize the system against external disturbances, which are broad in duration and repetition, the feedback should be multimodal, with paths that preferentially correct specific exacerbations. In the case of EPS, the fast correction of the null position is implemented via the *R*_1_ and *C* elements that ensure the fast recovery via velocity-type correction, which means that the disturbance is compensated with the derivative of the error signal. This type of velocity feedback, although effective in suppressing the short period cycling, neglects in principle the long period cycling and the permanent offsets of the loop where the velocity tends to be zero. This is an unwanted condition that can gradually saturate the system. In the case of MPS, the compensation of fast-cycled mood disturbances (ultracycling) is achieved by the administration of antiepileptic drugs (i.e., valproate or carbamazepine), which have been found to be effective in clinical trials. On the other hand, the effectiveness of lithium in regulating the fast component of bipolarity is limited. The fast correction in both the models is dominant and aggressive to overcome the severity and the acute character of the disturbance. The velocity-type correction, in the case of the MPS is achieved by evaluating mostly the velocity of the incoming cycling episodes or how fast is developed and less the local extreme strength of the episode itself. According to this notion, the optimal regulation of bipolar episodes is achieved by frequent patient evaluation and subsequent treatment manipulations. The integrator used in the EPS enhances the long period or semi-permanent deflections of the mass and effectively contributes to the long-term compensation of EPS. In the case of MPS, lithium in clinical trials has been found to be effective in blocking the slow cycling mood episodes, thus correcting the mood pendulum for either direction mania or depression. In neurobiological terms, lithium, by acting on second messenger systems, regulates neurotransmission of various systems, while by affecting neural developmental pathways increases cortical neuropil and neurogenesis acting thus as a prophylactic agent against future recurrences of the illness. The multiple time-dependent feedback corrections (electromechanical and pharmaceutical) should compensate simultaneously the fast as well as the slow cyclic component with effects that overlap to each other.

## Results

### Causes of instability during the correction

**Figure 2:**
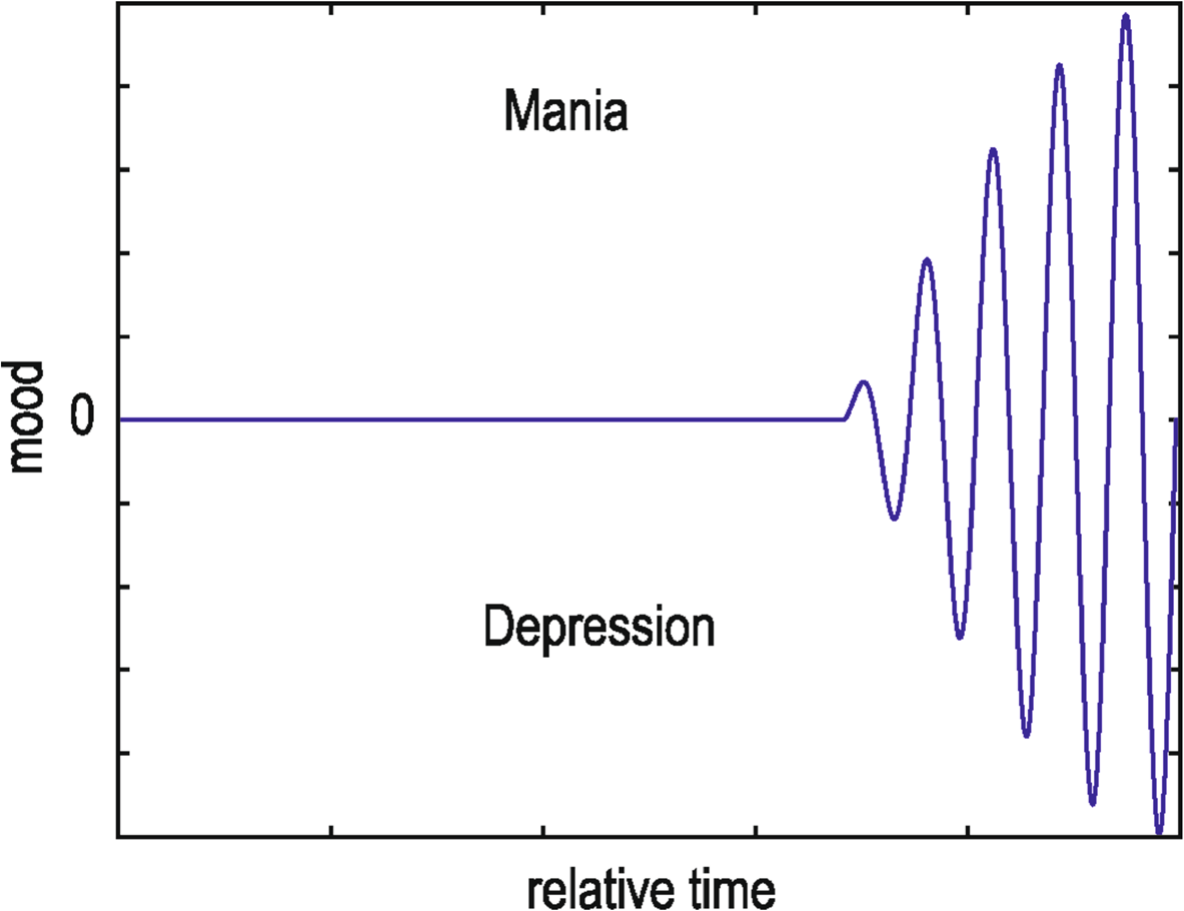
**Development of an oscillatory state caused by erroneous procedure in regulating an initial excitation.** Under this situation erroneous manipulation of the treatment renders the MPS inefficient to compensate the mood episodes.

Although the feedback correction is needed for the suppression of the oscillations in an excited system, the nature of which in the case of MPS has been speculated in the introduction; sometimes, the manipulations themselves could generate oscillations, and these are considered as feedback failures. More specifically, instabilities caused by overestimation are common situations where the applied correction is much stronger than the desired. Usually, embedded delays and errors in measuring the information regarding the deviations from the equilibrium lead both systems to overshoot and subsequently to oscillate. Data gathered from the mass model are illustrated in Figure 2. The impulse response of the system clearly shows the development of an oscillatory activity that is difficult to be compensated. Medication induced overcorrection usually derived from the administration of antidepressants (Ads) during the depressive phase of the illness or the use of neuroleptics (NLs) during the manic phase of the illness. Thus, in order to avoid this oscillatory attitude of the mood, Ads and NLs should be avoided. However, when these drugs are considered necessary, they should be administered over mood stabilizing medication (i.e., lithium or valproate).

**Figure 3:**
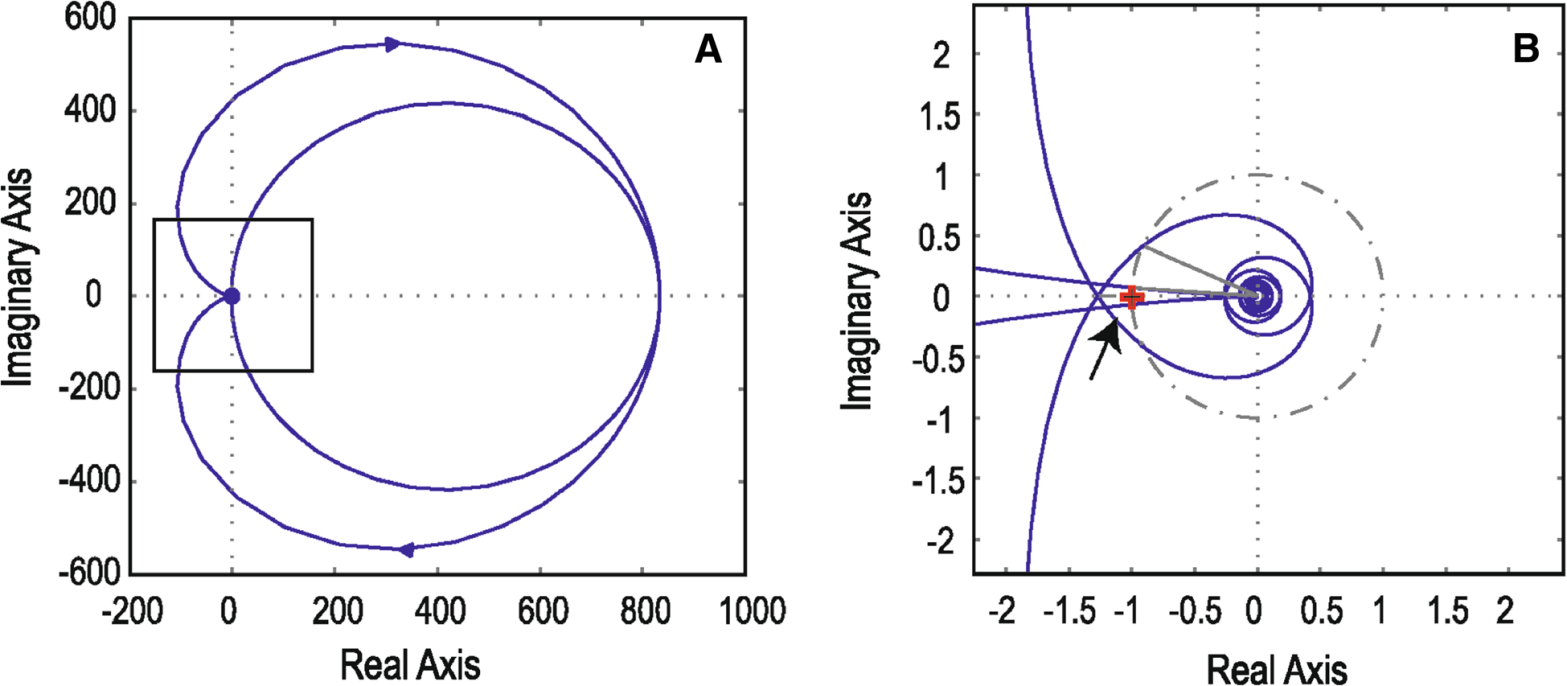
**The Nyquist plot corresponds to an unstable system. (A)** Nyquist plot indicating the stability margins of the mass pendulum system under unstable operation caused by a series of unexpected delays. **(B)** The plot focuses in the critical circle showing that −1 + j0 (red cross indicated by the arrow) is encircled by the trajectory line indicating thus the instability of the system. The multiple time-dependent feedback mechanism fails to recover the equilibrium.

**Figure 4:**
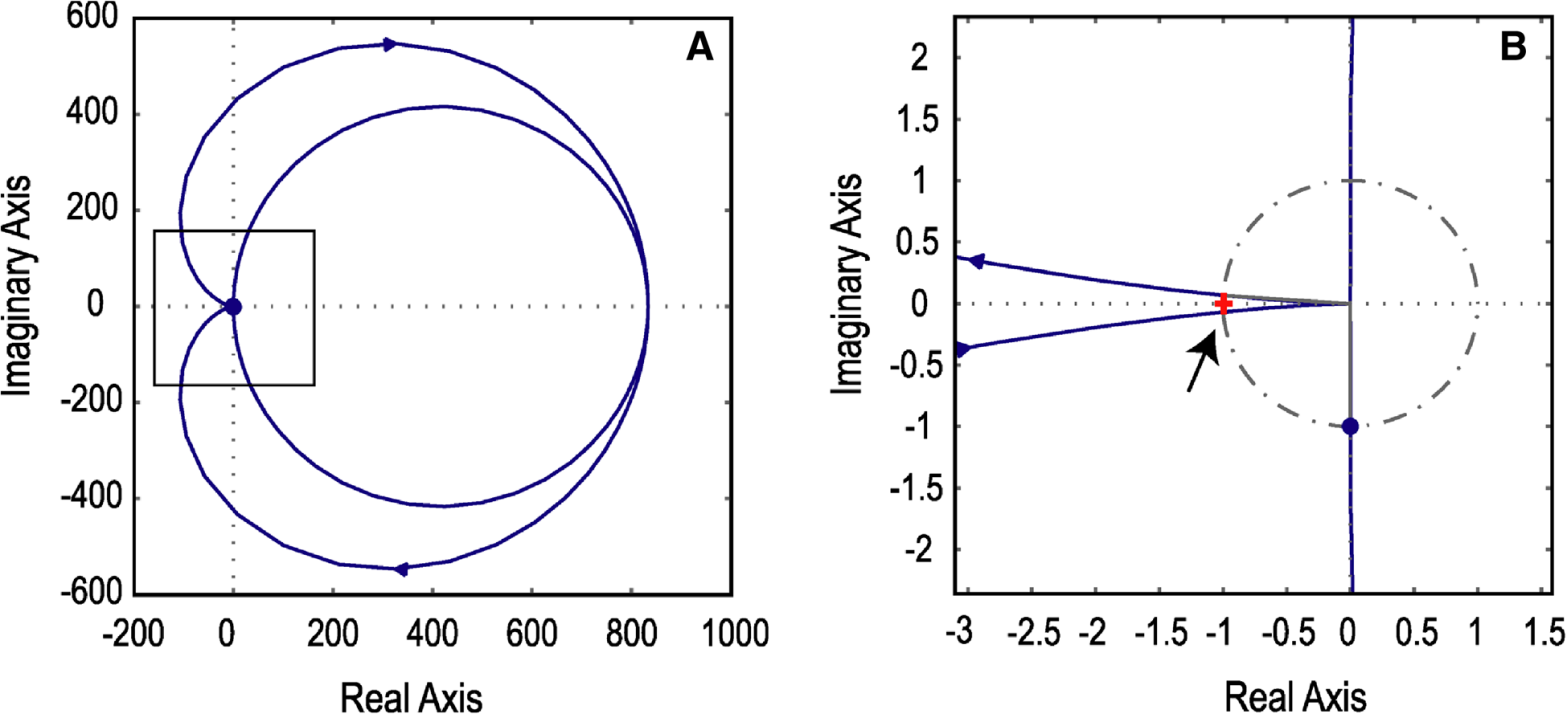
**The Nyquist plot corresponds to a stable and well compensated system. (A)** Nyquist plot indicating the stability margins of the mass pendulum system under stable operation with the −1 + j0 (red cross indicated by the arrow) not encircled by the trajectory line in plot **(B)** preconceiving thus a stable operation.

The introduction of unexpected input delays in the EPS render the system unstable and incapable to apply the proper feedback for the correction of the mass position. Figure 3A shows the Nyquist plot that examines the stability of the specific situation. The zoomed caption (Figure 3B) shows that the −1 + j0 point (noted with a red cross) is encircled, thus indicating that the system is unstable. Accordingly, in the level of mood disorder, delays between the diagnosis of the current state of the illness and the subsequent treatment commencement develop an inconsistent mode that induces instability and confusion in the therapeutic approach. Nyquist plot (Figure 4A) and the zoomed capture (Figure 4B) correspond to a stable and well-compensated system that ensures broadband stability against external excitations. The −1 + j0 point (noted with a red cross) is not encircled thus indicating stability. In both the abovementioned examples, the transfer function of the EPS is computed using the parameter values described previously (see ‘Stabilization of the inverted pendulum’ section). Although this parameter set ensures stability and high gain of the closed loop as shown in Figure 4, the introduction of an input delay is capable enough to destabilize an already stable system indicating the importance of phase errors during the correction.

**Figure 5:**
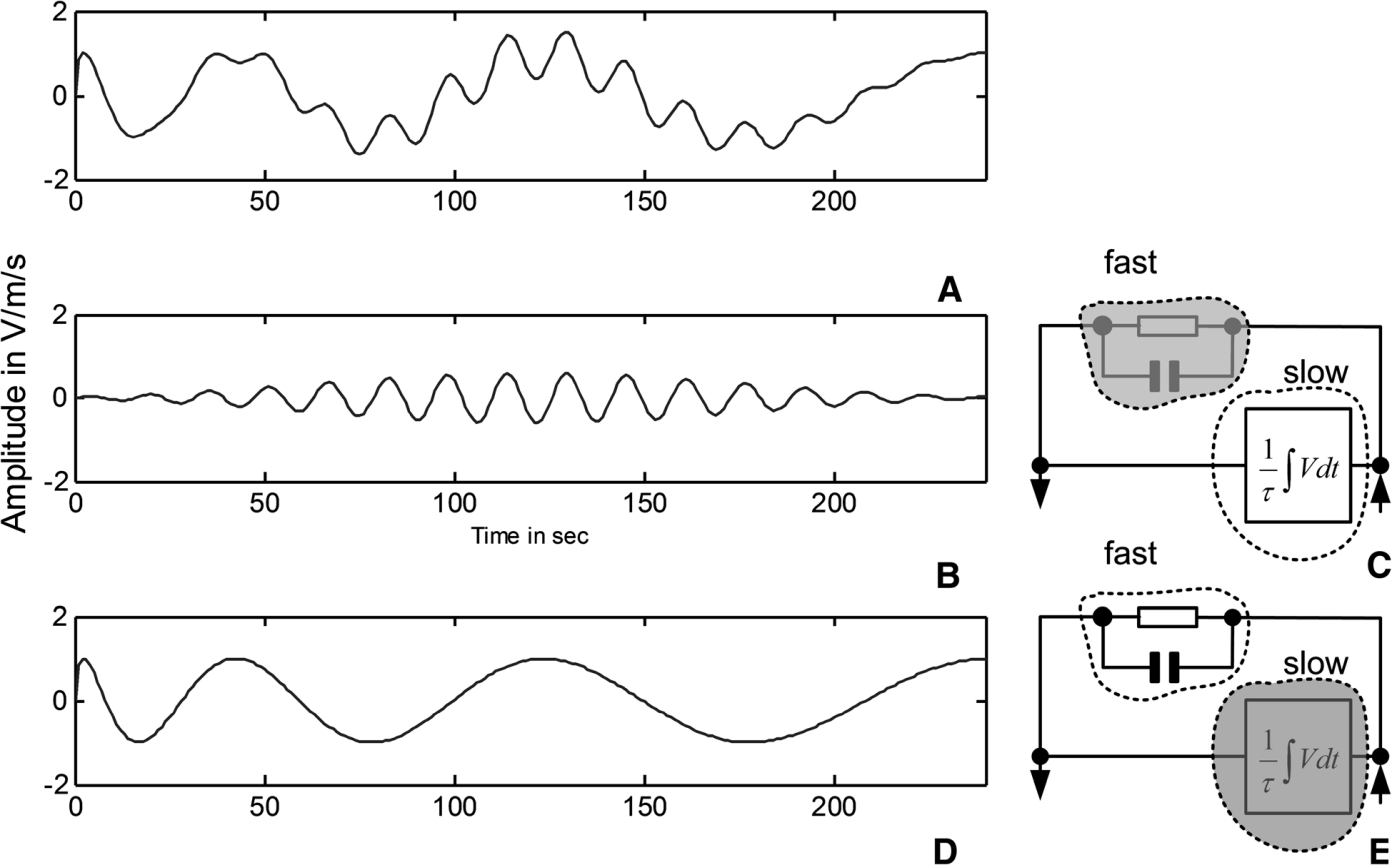
**Excitation of the inverted pendulum device installed under observatory grade conditions. (A)** A complex wave with broadband spectrum content (fast over slow) excites the mass of the EPS. **(B)** Oscillation of the mass of the EPS in the high-frequency component due to the lack of the fast feedback node. In this case, the long period excitation is effectively compensated. Shaded circle in **(C)** indicates the blocked fast feedback node. **(D)** Oscillation of the mass of the EPS in response to the long period component of the complex wave. Shaded circle in **(E)** indicates the blocked integrator of the feedback loop.

The importance of the frequency-time-selective compensation is cited in the following experimental trial where the inverted pendulum device, which is been installed at the Institute of Geodynamics of the National Observatory Athens, (Koutsoukos and Melis 2005) has been exposed in a broadband excitation for calibration purposes. Figure 5A shows the complex wave used to excite the mass of the pendulum. The lack of the feedback node (shaded circle) that covers the high-frequency stability allows the mass to oscillate at the high-frequency component of the excitation as shown in Figure 5B,C, while the long-period oscillation is effectively compensated. Exactly, the opposite happened when the integrative path of the feedback is blocked (shaded circle) as shown in Figure 5D,E. In this case, the system oscillates in response of the long-period excitation, while the fast component excitation is effectively compensated. At any case, the output of the experimental system is the mass position and its derivative in volts per meter per second (V/m/s). In general, by decreasing the current (strength) of a specific feedback node, the effect of the specific frequency zone is minimized. During the previous experimentation, the lack of the fast feedback component was achieved by increasing *R*_1_ in the range of several tenths of mega ohms (MΩ) and decreasing *C* in the range of picofarads (pF). In analogy, the lack of the integrative path was achieved by increasing *R*_2_ in the range of several tenths of MΩ.

**Figure 6:**
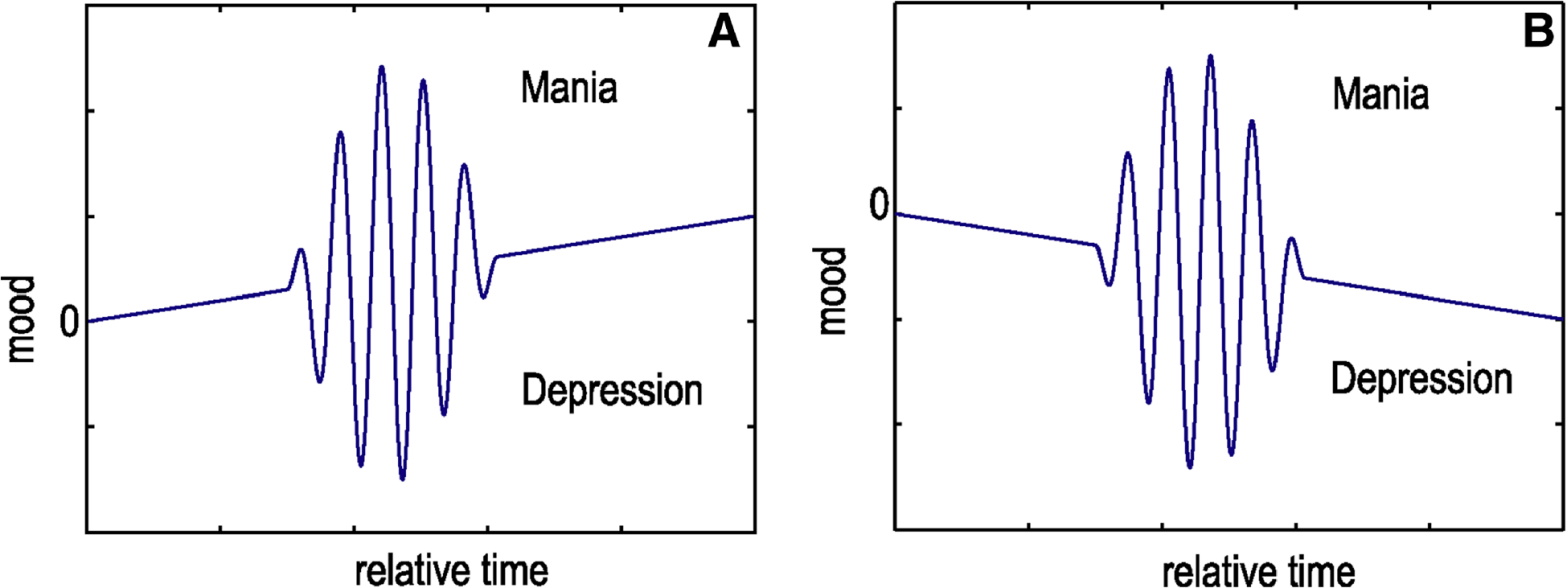
**Illustration of a complex mode.** Where mood oscillations have been developed over a long term (offset) that biases the system to either mania or depression **(A)** and **(B)**, respectively. This case demands a multimodal feedback to correct selectively excitations with different polarity and different time evolution.

A complex disturbance is illustrated diagrammatically in Figure 6A,B, representing the oscillatory attitude of the mood over a semi-permanent or long-term in declination to the opposite poles. The compensation in these cases demands a multimodal feedback to correct selectively excitations with different polarity and different time evolution. Following that, the contribution of the integrative path is substantial in regulating the system since the fast recovery path neglects in principle the slow declination of the system. This condition, in BDs, corresponds to fast mood transitions that overlap to hypomanic or depressive background. The use of a mood stabilizer (i.e., lithium), having integrative properties, is effective in treating slow mood fluctuations, while the co-administration of an antiepileptic drug compensates the fast mood oscillations ensuring a ‘broadband’ mood stabilization of the patient. Conclusively, the velocity-type correction that compensates effectively the mass motion in the level of the mood stabilization could be realized with treatment corrections in response to the rate of change (velocity) of the evolving mood episodes and not their polarity and strength.

## Discussion

In BD, a cyclical alternation between the two poles (mania and depression) is observed, with a periodicity measured in days, weeks, months, or years (Fisfalen et al. 2005; Goodwin and Jamison 2007). Typically, the ‘attacks’ of mania and/or depression in the natural evolution of the disease are highly irregular. The dynamical characteristics of bipolar illness raise the question, whether there is an underlying deterministic structure in the noisy natural trajectory of bipolar disorder. According to a speculation made from Globus and Arpaia (1994), a person with bipolar disorder would have two basins, one in the region of low motor activity with negative affect and the other in the region of high motor activity with positive affect. The height of the pass between these two basins would reflect the potential transition between the two extremes. The tendency of bipolar patients to evolve a more rapid cycling pattern over time could be attributed to a gradual lowering of the threshold between the two basins during the course of the illness.

We consider that energy is the common denominator of all processes. The energy of a process is its ability to change itself spontaneously and to produce change when it interacts with other processes. This consideration explains how physical and mental energy forms (i.e., mood, sleep, sexuality, etc.) interact in mood disorders, and in general, how biological, social, and psychological factors contribute in the causation of many medical and psychiatric illnesses. Under this view, bipolarity is a process that exchanges energy and this may be considered in the development of specific guidelines for the treatment of the illness. For example, there is evidence that the administration of tricyclic antidepressants increases the energy, accelerating the time course of bipolar fluctuations (Wehr and Goodwin 1979; Goodwin and Jamison 2007; Goldberg and Truman 2003) inducing in this way a switch to mania or a rapid cycling form of bipolar disorder. The energy exchange, equilibrium, feedback, stability, and oscillations are notions strongly interacting and exist in different ways in both EPS and MPS models.

In the present study, we examine and correlate the correction procedures that maintain the equilibrium of the mood, in the context that the involved actions exhibit functional similarities with the corrections applied to an electromechanical mass-pendulum system regulated by closed loop control. Although substantial differences exist between the ‘mass-motion’ and the ‘mood-motion’ substrates, the correction techniques applied in both systems exhibit a common mode of action, thus indicate possible similarities of the underlying mechanisms.

The nature of bipolar illness integrates multiple states where transient mood phases overlap on a permanent offset of the system that could be either depressive or manic. In these cases, the specific action of lithium behaves as an integrator and offers long-term stabilization during medication. Lithium, by acting on second messenger systems, regulates neurotransmission of various systems, contributes to the treatment of the current mood episode, while by affecting neurodevelopmental pathways increases cortical neuropil and neurogenesis affecting the recurrences of the mood episodes, providing thus mood stabilization. In correlation with the mass pendulum system, this action of lithium having the place of an integrator can contribute to the long-period maintenance of the equilibrium. In addition to this, the node of fast activity compensator is implemented by the administration of specific antiepileptic drugs that control the fast mood oscillations (ultra cycling, mixed type) of the bipolar illness. The degree of which the correction treatments applied in the two models was found functionally overlapped is in accordance with the current opinion, regarding the feedback control, which is that equilibria of complex dynamical systems are able to be maintained by the contribution of different procedures that compensate the excited system adaptively in different time scales.

Definitely, the processes involved in bipolar illness are more complex since the psycho-biological substrate incorporates parameters that cannot be defined and specific interactions that are distant from any generalization. Nevertheless, the conclusions extracted from the simpler model regarding the feedback applied in the mass-pendulum could influence the main direction of the treatment manipulations applied in the case of the bipolar illness. On the other hand, manipulations made on the transfer function of the mass-pendulum system allowed the identification of critical conditions that induce instability of the closed loop. These conditions were examined as potential causations for similar behavior observed in the mood system.

## Conclusions

In our study, we worked, from the one hand, with a fine instrument that effectively compensates the velocity of the ground motion (ground oscillations) and, from the other hand, with a distributed oscillating system that considers the mood as outcome of the psychic process, respectively. Although the transfer function and the stability mathematics describe in practical terms the functioning of the EPS, the lack of reliable long-term time series data, in the case of the mood description, limits the direct applicability of this approach in the form of strict numerical model. Thus, we stand in a more descriptive and less quantitative approach that ‘plates’ already followed guidelines for biological treatment of BDs (Grunze et al 2010) with the notions of feedback control. Particularly, the introduced velocity-type correction may contribute in developing treatment strategies to avoid treatment-emergent affective switches to mania and hypomania during the application of antidepressants in the treatment of bipolar depression. Additionally, the notion of multimodal feedback to correct selectively excitations with different polarity and different time evolution scales increases the effectiveness of the treatment approach.
